# Irisin treatment improves healing of dystrophic skeletal muscle

**DOI:** 10.18632/oncotarget.21636

**Published:** 2017-10-06

**Authors:** Musarrat Maisha Reza, Chu Ming Sim, Nathiya Subramaniyam, Xiaojia Ge, Mridula Sharma, Ravi Kambadur, Craig McFarlane

**Affiliations:** ^1^ School of Biological Sciences, Nanyang Technological University, Singapore; ^2^ Singapore Institute for Clinical Sciences (A^*^STAR), Brenner Centre for Molecular Medicine, Singapore; ^3^ Department of Biochemistry, YLL School of Medicine, National University of Singapore, Singapore; ^4^ Currently not affiliated with Department of Biochemistry, YLL School of Medicine, National University of Singapore, Singapore; ^5^ Currently not affiliated with School of Biological Sciences, Nanyang Technological University, Singapore; ^6^ Current/Present address: Department of Molecular & Cell Biology, College of Public Health, Medical and Veterinary Sciences, James Cook University, Townsville, QLD, Australia

**Keywords:** skeletal muscle, dystrophy, FNDC5, irisin, sarcolemmal stability

## Abstract

**Background:**

Irisin is an exercise induced myokine that is shown to promote browning of adipose tissue and hence, increase energy expenditure. Furthermore, our unpublished results indicate that Irisin improves myogenic differentiation and induces skeletal muscle hypertrophy. Since exercise induced skeletal muscle hypertrophy improves muscle strength, we wanted to investigate if ectopic injection of Irisin peptide improves skeletal muscle function in a mouse model of muscular dystrophy. This utility of Irisin peptide is yet to be studied in animal models.

**Methods:**

In order to test this hypothesis, we expressed and purified recombinant murine Irisin peptide from *E. coli*. Three- to six-week-old male *mdx* mice were injected IP with either vehicle (dialysis buffer) or Irisin recombinant peptide for two or four weeks, three times-a-week.

**Results:**

Irisin injection increased muscle weights and enhanced grip strength in *mdx* mice. Improved muscle strength can be attributed to the significant hypertrophy observed in the Irisin injected *mdx* mice. Moreover, Irisin treatment resulted in reduced accumulation of fibrotic tissue and myofiber necrosis in *mdx* mice. In addition, Irisin improved sarcolemmal stability, which is severely compromised in *mdx* mice.

**Conclusion:**

Irisin injection induced skeletal muscle hypertrophy, improved muscle strength and reduced necrosis and fibrotic tissue in a murine dystrophy model. These results demonstrate the potential therapeutic value of Irisin in muscular dystrophy.

## INTRODUCTION

Duchenne Muscular Dystrophy (DMD) is a common form of muscle wasting seen in young boys and is caused by mutations in the *dystrophin* gene [1]. Mutation of the *dystrophin* gene results in the production of a defective dystrophin protein leading to membrane fragility and muscle necrosis [2]. The functionality of muscle deteriorates in muscular dystrophy patients over time due to repeated cycles of skeletal muscle degeneration and regeneration, leading to muscle weakness [2]. Moreover, myofibers are progressively replaced by collagen, which promotes fibrosis and weakening of muscle. [3]. In the late stages of muscular dystrophy, patients become wheelchair bound since activities like walking become challenging and patients often die prematurely due to heart and respiratory failure [4]. Currently there are no known therapies to overcome DMD or any form of muscular dystrophy.

The naturally occurring *mdx* mouse model of DMD [5] is one of the most widely used models to study the pathophysiology of DMD. During the early stage of the disease, at 3 weeks of age, *mdx* mice undergo a sudden onset of muscular necrosis [6]. This acute onset of muscular dystrophy provides a good platform to investigate potential therapeutic molecules, which could alleviate this condition by reducing necrosis and improving muscle strength. *Mdx* muscle growth is slower than that of the wild type controls and hence, they have smaller fibers, myonuclear domains and less myonuclei [7]. Three week old *mdx* mice undergo severe muscle fiber destruction and subsequent complete regeneration, which leads to muscle fiber necrosis [8]. After 8 weeks of age, the extent of muscle fiber damage decreases and stabilizes despite repeated rounds of fiber necrosis and regeneration [8]. By 12 to 14 weeks of age, *mdx* mice attain the maximum size of muscle fibers, which are hyper-nucleated with more centrally placed nuclei [7].

Irisin is a newly discovered myokine that is induced in response to exercise [9]. The 112 amino acid protein Irisin, is formed through processing of the precursor protein FNDC5. Upon cleavage, mature Irisin protein is secreted into circulation and has been shown to induce the expression of genes that promote browning of beige adipose tissue, such as Ucp-1, Cidea, Cpt1b and Dio2 [9]. The effect of Irisin in mouse models is distinct and well established. However, the role of Irisin in humans remains controversial. Although Boström *et al*., (2012) demonstrated that exercise resulted in increased serum Irisin levels in humans, more recent work from Raschke *et al.*, (2013) revealed the absence of a canonical translational start codon in human Irisin, which indicated the production of a non-functional Irisin protein in humans [10]. Moreover, a separate study of commercially available antibodies by Albrecht *et al*., (2015) provided evidence to suggest that all commercial antibodies, available at the time, were unable to detect circulating Irisin protein in humans [11]. However, recent work by Jedrychowski *et al*, (2015) revealed that although human Irisin protein contains a non-canonical start codon, the full length Irisin precursor protein FNDC5 is translated, indicating complex regulation of translation. FNDC5 protein is present in human skeletal muscle and is cleaved to give rise to detectable levels of Irisin in circulation. In fact, the level of Irisin in circulation is more abundant than other commonly known circulating proteins, such as Insulin and Interleukin 6 (IL6). In addition, the authors showed that Irisin levels are elevated in response to exercise in humans and is indeed an exercise induced myokine as previously reported by Boström *et al*., (2012) [12].

In humans, circulating Irisin levels are positively correlated with biceps circumference and expression of IGF-1 [13]. This suggests that elevated levels of Irisin may lead to increased skeletal muscle mass. In agreement with this, elevated Irisin levels have been observed in *myostatin*-null mice, which display a dramatic double muscled phenotype [14]. Recently, it was further shown that Irisin is able to stimulate muscle growth-related genes in humans [15]. In human myocytes, Irisin secretion has been shown to increase during myogenic differentiation and Irisin treatment resulted in increased p-Erk expression, which is a key protein involved in the protein synthesis pathway [15]. Consistent with these results, recent unpublished data from our laboratory demonstrate that Irisin improves myogenic differentiation of C2C12 myoblasts and induces significant muscular hypertrophy when injected into wild type C57BL6/J mice. Taken together these studies reveal that increased levels of Irisin could promote skeletal muscle growth.

To study the potential therapeutic role of Irisin in increasing skeletal muscle growth, we investigated the effect of recombinant Irisin protein on skeletal muscle mass, quality and function in the *mdx* mouse model of DMD. Our results strongly suggest that Irisin is able to improve skeletal muscle mass and strength of dystrophic *mdx* mice, reduce fibrotic tissue accumulation as well as reduce necrosis of muscle fibers. Moreover, Irisin is able to protect dystrophic muscle from sarcolemmal instability, which could be indicative of enhanced muscle function in *mdx* mice. These data underscore the therapeutic potential of Irisin in improving skeletal muscle mass and protecting against dystrophic skeletal muscle loss.

## RESULTS

### Irisin injection promotes muscular hypertrophy in young *mdx* mice

**Figure 1:**
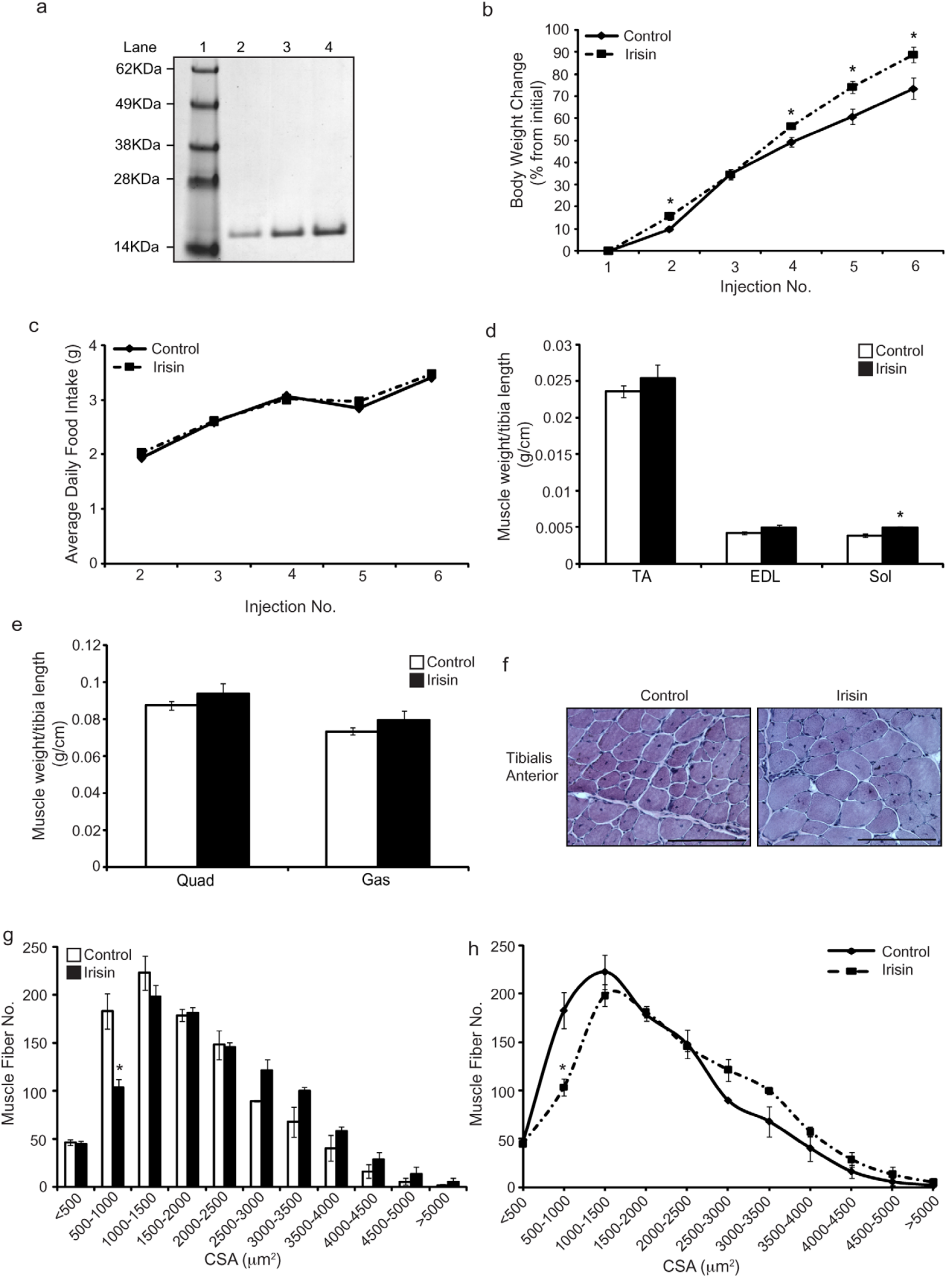
Irisin injection increases skeletal muscle mass and promotes hypertrophy in young *mdx* mice **(a)** Representative image of Coomassie stained protein gel displaying purified recombinant His-tagged Irisin protein. A single band at ~15kDa was detected for recombinant Irisin protein. Lane 1 shows the SeeBlue Plus 2 Pre-Stained ladder. Lanes 2, 3 and 4 show 1μg, 2μg and 3μg of the purified His-tagged Irisin protein, respectively. **(b)** Graph showing percentage change in body weight of young (3-week-old) male *mdx* mice injected three times-a-week with either DB or Irisin (2.5μg/gram body weight) for two weeks. Body weights were measured prior to each injection. **(c)** Graph showing average daily food intake per mouse. Food intake was measured prior to each injection over the two-week trial. **(d)** Graph showing the average weight of TA, EDL and Sol skeletal muscle tissues, normalized to tibia length (g/cm), from young *mdx* mice injected with either DB or Irisin for two weeks. **(e)** Graph showing the average weight of Quad and Gas skeletal muscle tissues, normalized to tibia length (g/cm), from young *mdx* mice injected with either DB or Irisin for two weeks (n=5 mice per group for all experiments above). **(f)** Representative images of Haematoxylin and Eosin (H&E) stained TA muscle cross sections obtained from young *mdx* mice injected with either DB or Irisin for two weeks. Images were captured using a 20x objective. Scale bar represents 100μm. **(g-h)** Graphs showing the distribution of TA myofiber CSA from young *mdx* mice injected with either DB or Irisin for two weeks. (n=3 mice per group). Error bars represent mean ± SEM. For all relevant figures, the appropriate statistical analysis was performed and significance is indicated with ^*^ (P<0.05).

SDS-PAGE analysis showed that the Histidine-tagged Irisin protein purified from *E.coli* is a homogenous protein (Figure 1a). Young (3-week-old) *mdx* mice were injected with either dialysis buffer (DB) or Irisin. Irisin injected mice showed a significant increase in the percentage change in body weight just after one week of Irisin injection (Injection 4). The significant increase in body weight was further maintained throughout the trial (Figure 1b). No distinct difference in food intake was noted between DB and Irisin injected mice (Figure 1c). Analysis of hind limb muscle tissue weights revealed an increase in the weights of all muscles analyzed, although with the exception of *M. soleus* (Sol) muscle, the increase was not statistically significant (Figure 1d and 1e). Histological analysis on transverse sections of the *M. tibialis anterior* (TA) muscle revealed hypertrophy of muscle fibers (Figure 1f); with an increased proportion of fibers with larger cross sectional area (>2500μm^2^) and a reduced proportion of fibers with smaller cross sectional area (<2500μm^2^) (Figure 1g and 1h).

### Irisin injection protects young *mdx* mice from dystrophic myofiber damage

**Figure 2:**
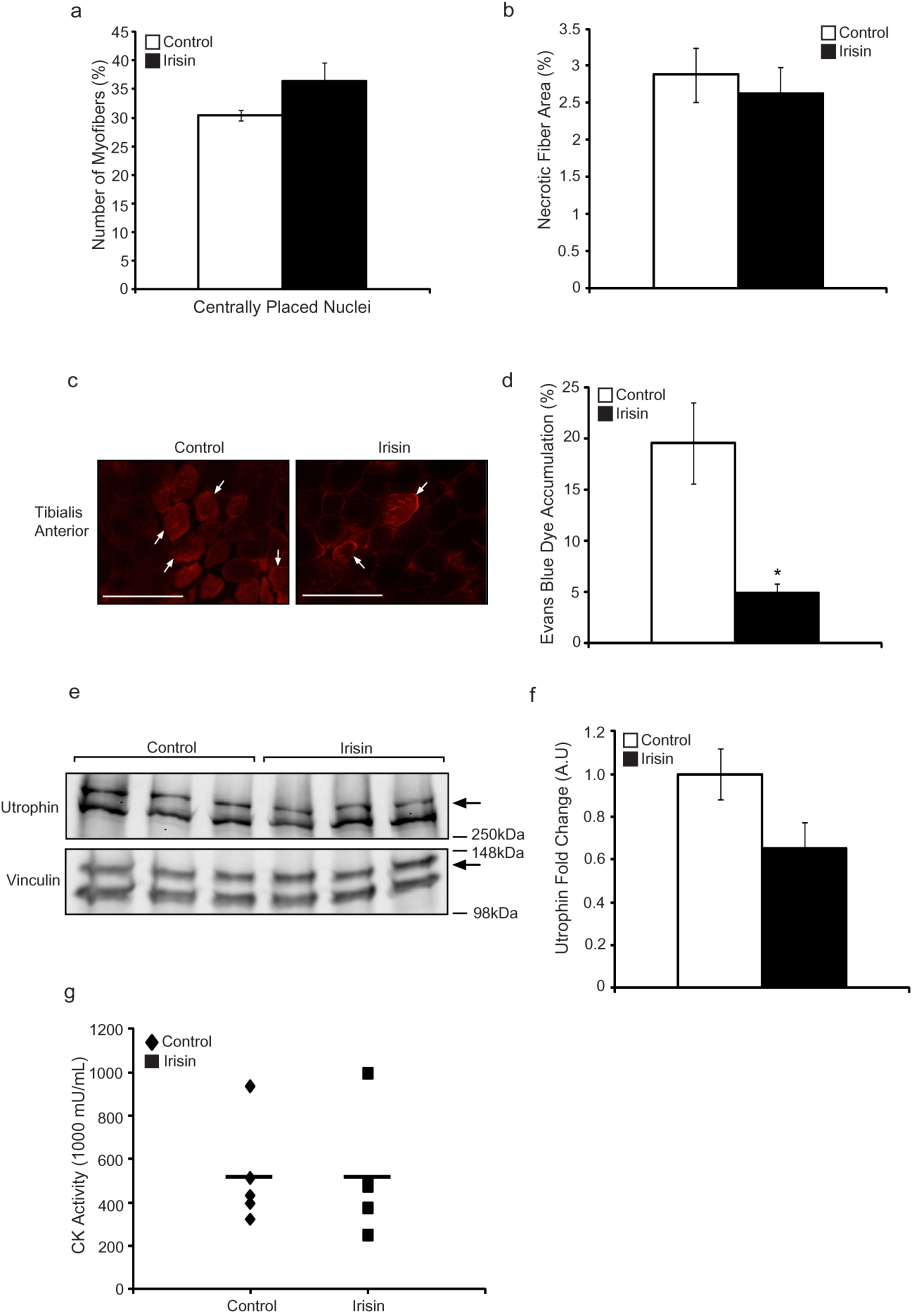
Irisin injection protects young mdx mice from dystrophic-associated skeletal muscle degeneration **(a)** Graph showing the percentage of myofibers with centrally placed nuclei in TA muscle from young *mdx* mice injected with either DB or Irisin for two weeks. **(b)** Graph showing the percentage of necrotic fiber area in TA muscle from young *mdx* mice injected with either DB or Irisin for two weeks. **(c)** Representative images of Evans blue dye (EBD) stained TA muscle cross sections obtained from young *mdx* mice injected with either DB or Irisin for two weeks. White arrows indicate a selection of representative EBD positive myofibers. EBD positive myofibers were visualized using fluorescent microscopy under a 20x objective. Scale bar represents 100μm. Linear brightness and contrast of images was adjusted to visualize EBD positive myofibers without altering the interpretation of results in any way. **(d)** Graph showing the percentage of EBD incorporation in TA muscle of young *mdx* mice injected with either DB or Irisin for two weeks. **(e)** Western Blot analysis of Utrophin protein levels in Gas muscle isolated from young *mdx* mice injected with either DB or Irisin for two weeks. The levels of Vinculin were assessed as a loading control. **(f)** Graph showing densitometric analysis of Utrophin protein levels in arbitrary units (A.U), normalized to Vinculin (n=3 mice for all figures above). **(g)** Graph representing creatine kinase activity in serum of young *mdx* mice injected with DB or Irisin (n=5 mice for both groups). Error bars represent ± SEM. For all relevant figures, the appropriate statistical analysis was performed and significance is indicated with ^*^ (P<0.05).

Skeletal muscle in *mdx* mice undergoes constant cycles of degeneration and regeneration. One of the hallmark features of newly formed (regenerated) muscle fibers is centrally placed nuclei. As such, we next quantified the number of myofibers with centrally placed nuclei in DB and Irisin injected mice, to assess if Irisin treatment improves postnatal myogenesis in *mdx* mice. Our analysis revealed a ~20% increase (albeit not statistically significant) in the percentage of myofibers with centrally placed nuclei in mice injected with Irisin, when compared to DB controls (Figure 2a). The percentage of necrotic muscle fibers was also quantified between DB and Irisin injected *mdx* mice, as assessed through the presence of infiltrating mono-nucleated cells, myofibers with fragmented sarcoplasm and hyper-contracted myofibers. Subsequent quantification revealed a 9% reduction in necrotic myofibers in *mdx* mice injected with Irisin, when compared to DB treated controls (Figure 2b); however, the reduction was not found to be statistically significant (Figure 2b). We further injected Evans Blue Dye (EBD) into DB and Irisin injected *mdx* mice (Figure 2c) to assess myofiber permeability. Red fluorescent EBD positive (Figure 2c) myofibers were assessed and the percentage of fluorescent muscle fibers was quantified through microscopy. Results revealed a significant 75% reduction in red fluorescent fibers in the TA muscles of *mdx* mice injected with Irisin, when compared to DB injected controls (Figure 2d). The level of Utrophin, a homologue of dystrophin, which is expressed in dystrophic muscle, was assessed in *M. Gastrocnemius* (Gas) muscle following two weeks of Irisin injection. Utrophin expression was normalized to the housekeeping protein, Vinculin. Results revealed a slight reduction, in the levels of Utrophin in *mdx* mice injected with Irisin, when compared to DB injected controls, albeit not statistically significant (Figure 2e and 2f). We next measured the creatine kinase (CK) activity in the serum of the *mdx* mice, which is used as a marker to identify the extent of muscular dystrophy, since increased CK level is associated with muscular dystrophy. However, we did not observe a distinct difference in CK activity in serum collected from *mdx* mice injected with either DB or Irisin (Figure 2g). Taken together, these data reveal that Irisin treatment is able to, at least in part, alleviate the dystrophic phenotype in young *mdx* mice.

### Irisin promotes skeletal muscle hypertrophy and protects against fibrosis, myofiber necrosis and sarcolemmal instability in adult *mdx* mice

**Figure 3:**
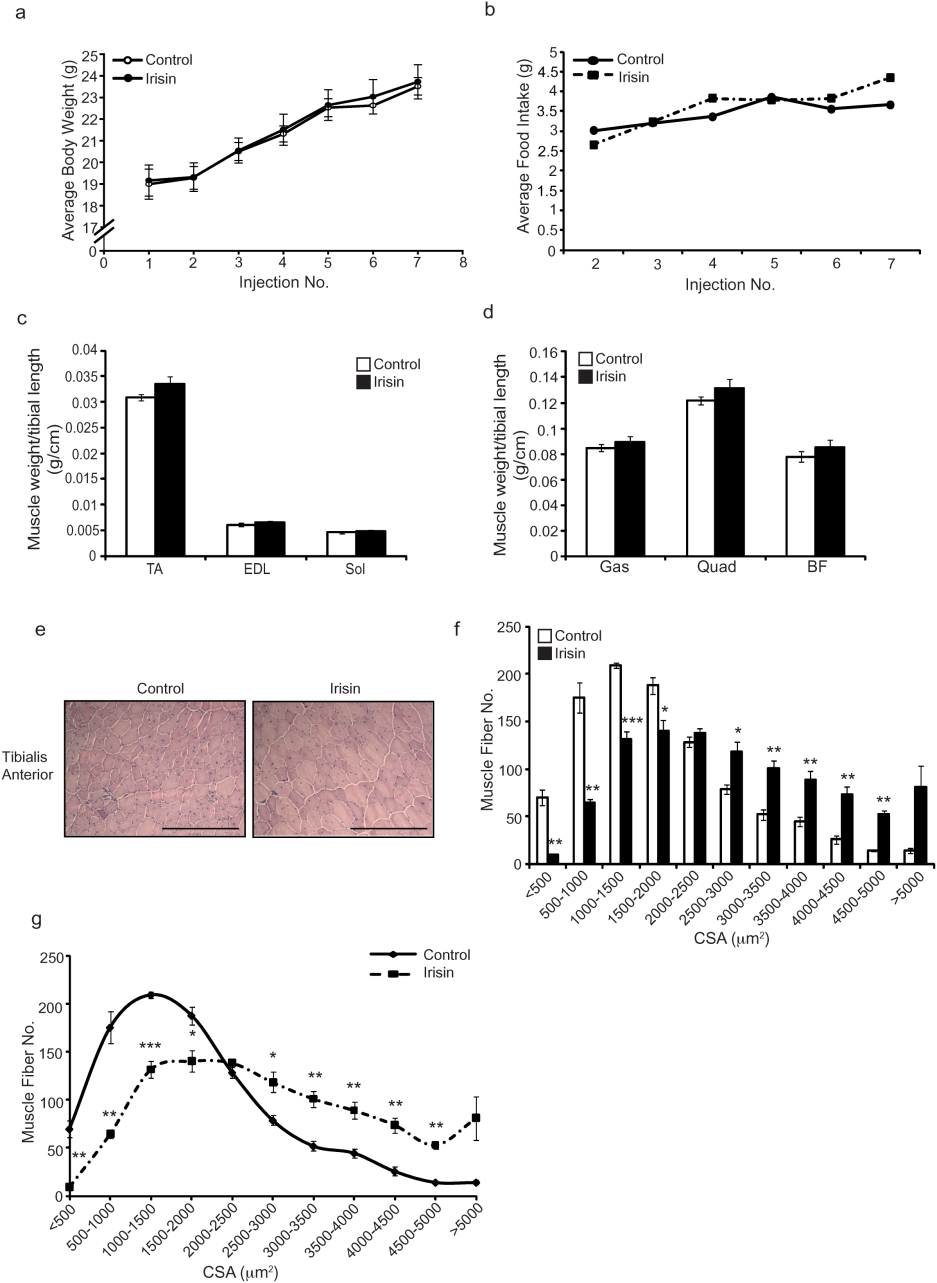
Irisin injection enhances muscle weights and promotes skeletal muscle hypertrophy in adult *mdx* mice **(a)** Graph showing average body weights of adult (6-week-old) male *mdx* mice injected three times-a-week with either DB or Irisin (2.5μg/gram body weight) for two weeks. Body weights were measured prior to each injection. **(b)** Graph showing average daily food intake per mouse. Food intake was measured prior to each injection over the two-week trial. **(c)** Graph showing the average weight of TA, EDL and Sol skeletal muscle tissues, normalized to tibia length (g/cm), from adult *mdx* mice injected with either DB or Irisin for two weeks. **(d)** Graph showing the average weight of Gas, Quad and BF skeletal muscle tissue, normalized to tibia length (g/cm), from adult *mdx* mice injected with either DB or Irisin for two weeks (n=6 mice per group for all experiments above). **(e)** Representative images of Haematoxylin and Eosin (H&E) stained TA muscle cross sections obtained from adult *mdx* mice injected with either DB or Irisin for two weeks. Images were captured using a 20x objective. Scale bar represents 100μm. **(f-g)** Graphs showing the distribution of TA myofiber CSA from adult *mdx* mice injected with either DB or Irisin for two weeks (n=4 mice per group for all experiments above). Error bars represent mean ± SEM. For all relevant figures, the appropriate statistical analysis was performed and significance is indicated with ^*^ (P<0.05), ^**^ (P<0.01) and ^***^ (P<0.001).

In order to determine the therapeutic potential of Irisin in alleviating the dystrophic phenotype observed in adult *mdx* mice, 6-week-old (adult) *mdx* mice were injected with either DB or Irisin three times-a-week for two weeks. No significant difference in body weight (Figure 3a) or food intake (Figure 3b) was noted between DB and Irisin injected adult *mdx* mice. However, consistent with results noted upon injection of young *mdx* mice with Irisin, an increase in weights of TA, *M. extensor digitorum longus* (EDL), Sol, *M. quadricep*s (Quad) and *M. biceps femoris* (BF) muscles was seen in adult mice injected with Irisin, although the increase in muscle weights was not statistically significant (Figure 3c and 3d). Histological analysis on transverse sections of the TA muscle revealed hypertrophy of muscle fibers (Figure 3e). Specifically, an increased proportion of fibers with larger cross sectional area (>2000μm^2^) and a reduced proportion of fibers with smaller cross sectional area (<2000μm^2^) was observed in Irisin injected adult *mdx* mice, when compared to DB injected controls (Figure 3f and 3g). It is noteworthy to mention that TA muscles from adult *mdx* mice injected with Irisin had a large number of myofibers with very large cross sectional area (>4000μm^2^), when compared to DB injected controls (Figure 3f and 3g). These data strongly suggest that Irisin promotes significant skeletal muscle hypertrophy in adult *mdx* mice. It is also important to mention that two weeks of Irisin treatment resulted in a more profound hypertrophy of skeletal muscle in adult *mdx* mice, when compared to young mdx mice (Compare Figure 1g and 1h with Figure 3f and 3g).

**Figure 4:**
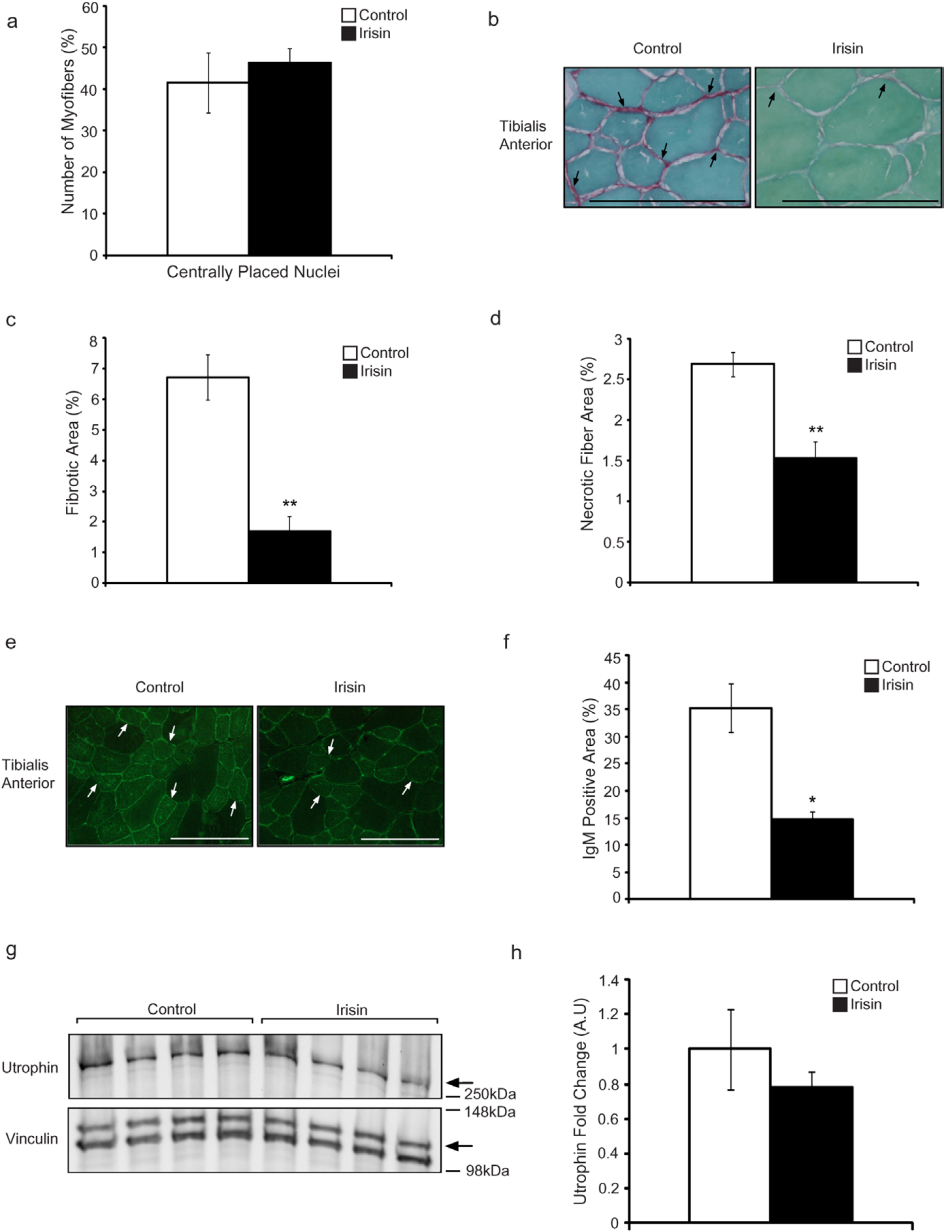
Irisin protects adult mdx mice from fibrotic tissue accumulation and muscle fiber degeneration **(a)** Graph showing the percentage of myofibers with centrally placed nuclei in TA muscle from adult *mdx* mice injected with either DB or Irisin for two weeks (n=4 mice for both groups). **(b)** Representative images of Sirius red and Fast green stained TA muscle cross sections obtained from adult *mdx* mice injected with either DB or Irisin for two weeks. The green stain indicates non-collagenous proteins, while the red stain indicates collagen. Black arrows indicate a selection of representative fibrotic areas. Images were captured using a 40x objective. Scale bar represents 100μm. **(c)** Graph showing fibrotic area (red stain), expressed as a percentage of total muscle section area, in TA muscle from adult *mdx* mice injected with either DB or Irisin for two weeks, as assessed through Sirius red and Fast green staining. **(d)** Graph showing the percentage of necrotic fiber area in TA muscle from adult *mdx* mice injected with either DB or Irisin for two weeks (n=3 mice per group for all experiments above). **(e)** Representative images of IgM antibody stained TA muscle sections obtained from adult *mdx* mice injected with either DB or Irisin for two weeks. White arrows indicate a selection of representative IgM positive myofibers. Images were captured using a 20x objective. Scale bar represents 100μm. Linear brightness and contrast of images was adjusted to visualize IgM positive myofibers without altering the interpretation of results in any way. **(f)** Graph showing IgM stained area, expressed as a percentage of total muscle section area, in TA muscle sections from adult *mdx* mice injected with either DB or Irisin for two weeks. **(g)** Western Blot analysis of Utrophin protein levels in Gas muscle isolated from adult *mdx* mice injected with either DB or Irisin for two weeks. The levels of Vinculin were assessed as a loading control. **(h)** Graph showing densitometric analysis of Utrophin protein levels in arbitrary units (A.U), normalized to Vinculin (n=4 mice per group for all experiments above). Error bars represent mean ± SEM. For all relevant figures, the appropriate statistical analysis was performed and significance is indicated with ^*^ (P<0.05) and ^**^ (P<0.01).

To further understand the beneficial effects of Irisin on *mdx* muscle, we next analyzed the difference in the proportion of regenerating muscle fibers, as represented by the number of centrally placed nuclei, between adult *mdx* mice injected with either DB or Irisin. It is interesting to note that despite the increase in myofiber cross sectional area noted in the TA muscle from Irisin injected *mdx* mice, the proportion of myofibers with centrally placed nuclei was not significantly different (Figure 4a), with ~41% and ~46% of myofibers containing centrally formed nuclei in DB and Irisin injected *mdx* mice, respectively. To confirm that the increase in muscle weights was not due to increased accumulation of fibrotic tissue and collagen, we performed Sirius Red (collagen) and Fast green (non-collagenous proteins) staining of skeletal muscle sections from TA muscle of DB and Irisin injected *mdx* mice (Figure 4b). Subsequent quantification of the fibrotic area (red) revealed a significant ~4-fold reduction in fibrosis in TA muscle from Irisin injected *mdx* mice, when compared to DB injected mice (Figure 4c), suggesting that Irisin treatment protected dystrophic muscle fibers against fibrotic tissue accumulation.

We next assessed the necrotic muscle fiber area in muscles isolated from adult *mdx* mice injected with either DB or Irisin. Adult *mdx* mice injected for two weeks with Irisin displayed a dramatic 43% reduction in necrotic muscle fibers, when compared to respective DB controls (Figure 4d). Taken together these data reveal a role for Irisin in protecting against myofiber necrosis in adult *mdx* mice.

Myofibers in *mdx* mice have reduced sarcolemmal stability, which allows proteins that are usually found only in circulation to enter and accumulate in the degenerating muscle fibers. To understand the role of Irisin in protecting sarcolemmal integrity of muscle from *mdx* mice, we investigated the accumulation of IgM protein in myofibers, which is typically restricted to circulation in healthy mice. Subsequent analysis revealed that a large proportion of the myofibers from *mdx* mice injected with DB stained positively for IgM (Figure 4e). However, Irisin injection resulted in a significant 58% reduction in IgM accumulation in myofibers from *mdx* mice (Figure 4f). These data suggest that Irisin improves the sarcolemmal stability of dystrophic myofibers. Lastly, we investigated the levels of Utrophin in adult *mdx* mice injected with either DB or Irisin. Similar to the results obtained above (Figure 2e and 2f), we observed slight reduction in Utrophin levels in adult *mdx* Gas muscle (Figure 4g and 4h). However, the reduction was not statistically significant.

### Prolonged Irisin treatment increases muscle weights and enhances grip strength of *mdx* mice

**Figure 5:**
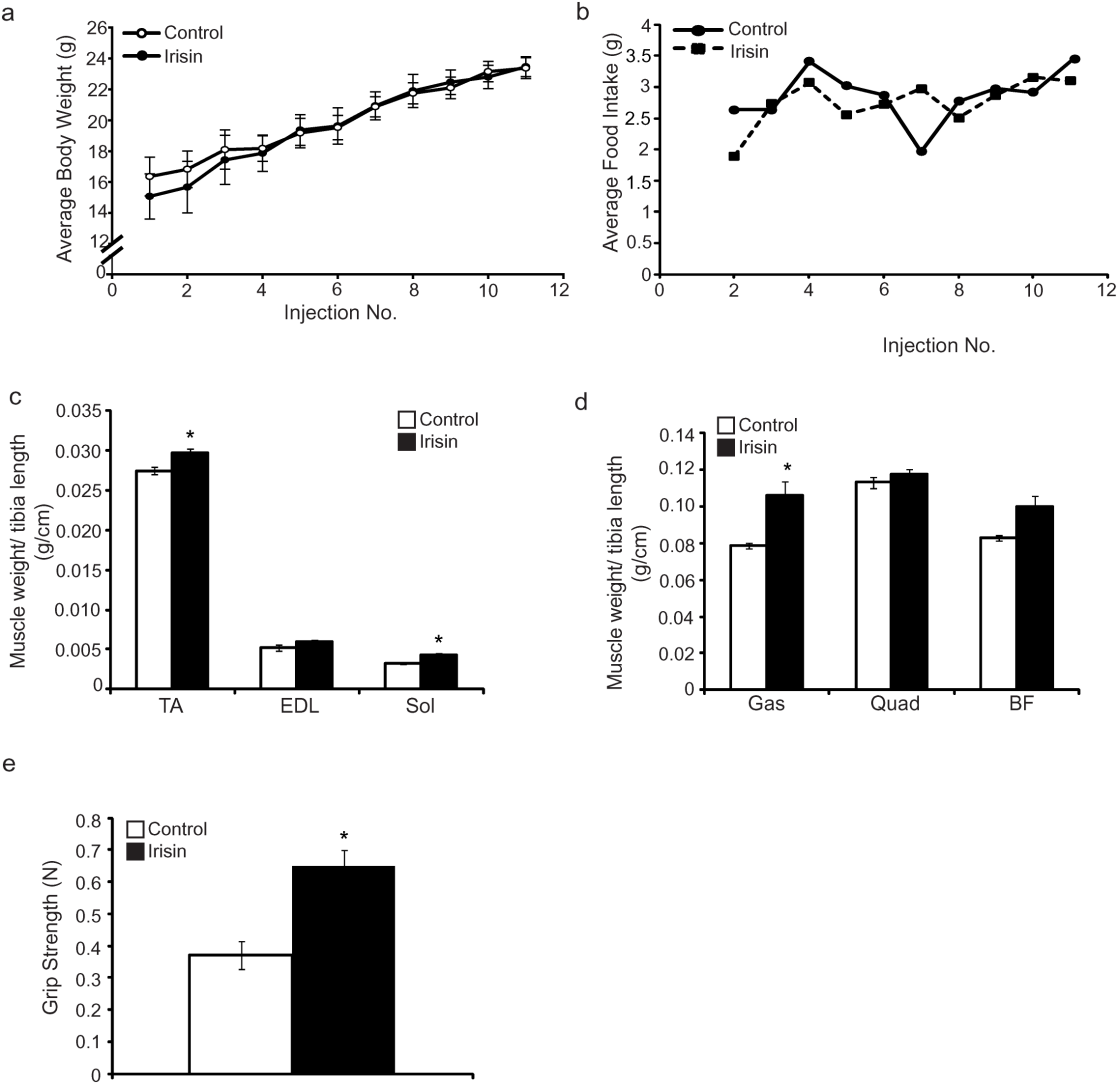
Prolonged Irisin injection increases skeletal muscle mass and enhances muscle function in *mdx* mice **(a)** Graph showing average body weight of 4-week-old male *mdx* mice injected three times-a-week with either DB or Irisin (2.5μg/gram body weight) for four weeks. Body weights were measured prior to each injection. **(b)** Graph showing average daily food intake per mouse. Food intake was measured prior to each injection over the four-week trial. **(c)** Graph showing the average weight of TA, EDL and Sol skeletal muscle tissues, normalized to tibia length (g/cm), from 4-week-old *mdx* mice injected with either DB or Irisin for four weeks. **(d)** Graph showing the average weight of Gas, Quad and BF skeletal muscle tissues, normalized to tibia length (g/cm), from 4-week-old *mdx* mice injected with either DB or Irisin for four weeks. **(e)** Graph showing the forelimb grip strength (N) of 4-week-old *mdx* mice injected with either DB or Irisin for four weeks. Grip strength was assessed prior to termination of the mice. (n=3 mice for the DB injected control group and n=4 mice for the Irisin injected group for all experiments above). Error bars represent mean ± SEM. For all relevant figures, the appropriate statistical analysis was performed and significance was indicated with ^*^ (P<0.05).

As described above, short-term (2 weeks) injection of Irisin into *mdx* mice tended to increase skeletal muscle weights, although the increase noted in muscle weights was not statistically significant. Given this, we next wanted to investigate whether or not increasing the duration of Irisin treatment of *mdx* mice could improve skeletal muscle weight and function. To study this, 4-week-old *mdx* mice were injected with either DB or Irisin, three times-a-week for 4 weeks. No significant difference in body weight (Figure 5a) or food intake (Figure 5b) was noted between *mdx* mice injected with either DB or Irisin for 4 weeks. However, analysis of individual hind limb skeletal muscle weights revealed a significant increase in TA, Sol and Gas muscle weights in Irisin injected *mdx* mice, when compared to DB injected controls (Figure 5c and 5d). The weights of EDL, Quad and BF skeletal muscles also showed an increase. However, this change was not statistically significant (Figure 5c and 5d). Importantly, 4 weeks of Irisin injection further resulted in a significant increase (~2-fold) in the forelimb grip strength of *mdx* mice (Figure 5e). These data indicate that prolonging the duration of Irisin treatment could improve both skeletal muscle mass and muscle strength of *mdx* mice.

## DISCUSSION

Irisin is a novel exercise-induced myokine and to date the role of Irisin on adipose tissue [16] and in type II diabetes [17, 18] has been researched extensively. Exercise is known to confer significant beneficial effects on skeletal muscle, especially through enhancing muscle strength through hypertrophy [19–22]. Since Irisin is a myokine and is found in significantly higher concentrations post-exercise [12], we reasoned that Irisin might function in an autocrine/paracrine manner to impart beneficial effects of exercise on skeletal muscle. Here we show that exogenous Irisin injection resulted in muscle hypertrophy and enhanced grip strength in dystrophic mice. Irisin treatment also reduced fibrosis and necrosis in dystrophic muscle fibers. Moreover, Irisin improved sarcolemmal integrity in the absence of dystrophin in *mdx* mice. Taken together, these data exemplify the therapeutic benefit of Irisin in improving muscle mass, muscle healing and muscle function in a dystrophic mouse model.

To assess the effect of Irisin on dystrophic muscle loss we have undertaken studies in both young and adult *mdx* mice. We have utilized *mdx* mice in the current study, as this model has been extensively used to study the pathophysiology of DMD [23]. Acute onset of the dystrophic phenotype, which includes myofiber necrosis and elevated creatine kinase levels, occurs in *mdx* mice at 3 weeks of age, with a gradual decrease in the intensity of the phenotype observed from 8 weeks of age onwards [8]. A low level chronic dystrophic pathology is maintained in *mdx* mice throughout their life, with a further reduction in severity noted after one year [24].

Initially, we assessed the effect of exogenous Irisin treatment on young (3-week-old) *mdx* mice undergoing acute dystrophic muscle loss. It is important to mention that we did not observe any adverse effects on the mice in response to Irisin injection. Parameters such as movement, behavior, or food consumption did not show significant differences after Irisin injection. The Irisin injected mice did not show inflammation, organ toxicity or fluid retention upon gross pathological examination. This indicated that the Irisin protein used for the experiments was safe for injection into the mice. Interestingly, while no apparent difference in food intake was noted between DB and Irisin injected mice (Figure 1c), a significant increase in percentage body weight change was observed in Irisin injected *mdx* mice (Figure 1b). We propose that the increased body weight was due in part, to increased muscle mass. Consistent with this, increased muscle weights (Figure 1d and 1e) (although not statistically significant) and mild myofiber hypertrophy was noted in three-week-old Irisin injected *mdx* mice (Figure 1f-1h).

Further results suggest that Irisin injection may have benefits in alleviating the dystrophic phenotype observed in skeletal muscle of *mdx* mice. A trend towards reduced myofiber necrosis was noted in young *mdx* mice following two weeks of Irisin injection, albeit not statistically significant (Figure 2b). During muscular dystrophy, the absence of dystrophin protein severely diminishes sarcolemmal stability and hence, proteins like albumin, which are otherwise restricted to circulation, are able to cross the sarcolemma and accumulate in myofibers [25]. Evans blue dye binds to serum albumin with high affinity and hence, Evans blue dye accumulation in skeletal muscle can be used as an accurate indicator of muscle damage [26] [27]. Importantly, a significant reduction in Evans blue dye accumulation was noted in myofibers of *mdx* mice injected with Irisin, which further suggests that Irisin may be able to protect myofibers from sarcolemmal damage during dystrophy (Figure 2c and 2d).

CK is an enzyme predominantly present in skeletal muscle, heart and the brain [28]. During muscular dystrophy, CK leaks out from skeletal muscle resulting in a spike in the levels of CK in circulation [23]. Despite the reduced myofiber necrosis and improved sarcolemmal integrity noted in *mdx* mice muscle following Irisin injection, no significant reduction in the levels of CK was observed in serum collected from Irisin injected *mdx* mice, when compared to DB injected controls (Figure 2g). We propose that the disparity between reduced necrosis/improved sarcolemmal integrity and the unaltered CK levels noted in serum may be due to the increased muscle mass observed in Irisin injected *mdx* mice. In other words, the increased skeletal muscle mass induced in response to Irisin injection may have masked the expected reduction in systemic CK activity. In agreement with this, a recent publication by Relizani *et al*., (2014) reported that blockade of Myostatin signaling in *mdx* mice, using a soluble form of the Activin Type II B receptor (sActRIIB), resulted in increased myofiber size in Soleus muscle without altering the overall levels of CK in serum [29]. Therefore, it is plausible that serum CK levels may not accurately reflect the extent of muscle damage present in Irisin injected *mdx* mice. However, it is quite possible that the protective role of Irisin was not sufficient to reduce the levels of serum CK in young *mdx* mice and perhaps prolonged Irisin treatment may have been required.

Similar to the changes in muscle mass noted in young Irisin injected *mdx* mice, injection of Irisin into adult *mdx* mice resulted in increased muscle weights, although the increase was not statistically significant (Figure 3c and 3d). However, with just two weeks of Irisin injection, a more pronounced hypertrophy of skeletal muscle was noted in Irisin injected adult *mdx* mice (Figure 3e-3g), when compared to young *mdx* mice (Figure 1f-1h). In dystrophic muscle, repeated rounds of degeneration and subsequent regeneration replace contractile muscle tissue with non-contractile fibrotic tissue, leading to a gradual loss of muscle strength in dystrophic muscle [30]. Consistent with improved muscle regeneration, Irisin injected *mdx* muscle had a pronounced reduction in fibrotic tissue accumulation (Figure 4b and 4c). Moreover, a significant, 43% reduction in necrotic muscle fibers (Figure 4d) was also observed after Irisin treatment, suggesting that injection of Irisin imparts protection against muscle degeneration and necrosis in adult *mdx* mice. It is important to mention that Irisin injection was able to reduce myofiber necrosis in adult *mdx* mice (~43%) (Figure 4d) to a greater extent than in young *mdx* mice (~9%) (Figure 2b), suggesting that Irisin effect may be more pronounced in adult *mdx* mice.

IgM is an antibody secreted by B cells and it is found mainly in circulation [31]. During muscular dystrophy, the sarcolemma of dystrophic muscle fibers become unstable and as such sarcolemmal integrity is compromised [32]. As a consequence of this, IgM, despite being a 900kDa molecule, is able to gain entry and accumulate in damaged myofibers [26]. A reduction of IgM accumulation in dystrophic muscle fibers is indicative of improvement of sarcolemmal stability [33]. Importantly, a distinct reduction in IgM accumulation was noted in myofibers of adult *mdx* mice injected with Irisin (Figure 4e and 4f), further validating the role of Irisin in maintaining sarcolemma integrity during dystrophy.

Utrophin is the autosomal paralogue of dystrophin and in fact has a similar function and structure to dystrophin [34]. Given this, Utrophin is able to compensate for the lack of dystrophin in dystrophic muscle and hence, the expression of Utrophin is typically higher in dystrophic conditions [35]. However, we did not observe a distinct difference in Utrophin levels in both young (Figure 2e and 2f) and adult *mdx* mice (Figure 4g and 4h), after injection with Irisin, although we did notice an increase in muscle mass (Figure 1d and 1e, Figure 3c and 3d), improved muscle strength (Figure 5e) and reduced fibrosis (Figure 4b and 4c). These data suggest that Irisin-mediated improvement of the muscle phenotype in *mdx* mice may not be due to Irisin signalling via Utrophin.

As alluded to above, Irisin injection was able to confer a greater protective effect on skeletal muscle in adult *mdx* mice, when compared to the young *mdx* mice. Notably, a more distinct increase in myofiber hypertrophy (Figure 3e-3g) and reduction in myofiber necrosis (Figure 4d) was observed in adult *mdx* mice after injection with Irisin. We propose that the difference in Irisin effect between young and adult *mdx* mice could be attributed to the natural stabilization of the dystrophic phenotype in adult mice [8, 24]. Hence, Irisin is able to exert a greater beneficial effect on muscle in adult *mdx* mice, which display low grade chronic pathology, as opposed to young *mdx* mice, which show more acute muscle damage [8, 24]. Importantly, prolonging the duration of Irisin injection in *mdx* mice resulted in a more pronounced increase in skeletal muscle mass and improvement of skeletal muscle function. These data underscore the pro-myogenic capacity of Irisin and further reveal that prolonged treatment with Irisin may impart greater improvement to *mdx* mice.

Previous work has revealed that loss of *myostatin* leads to increased expression of FNDC5 and Irisin [14]. Myostatin is a potent negative regulator of skeletal muscle mass and importantly, inhibition of Myostatin has been shown to reduce the severity of dystrophy, increase muscle strength [36] and reduce fibrosis in *mdx* mice [37]. Therefore we propose that Irisin may play a role in the enhanced muscle growth and reduced fibrosis noted in *mdx* mice in response to loss of myostatin. In agreement with this, we have shown that Irisin not only reduces fibrotic tissue accumulation (Figure 4b and 4c), but also improves muscle strength (Figure 5e) and skeletal muscle hypertrophy in *mdx* mice (Figures 1g and 1h, Figure 3f and 3g).

IGF-1 has been shown to rescue muscle loss in *mdx* mice and significantly reduce fibrosis [3]. Recent studies have revealed that treatment with Irisin results in upregulation of IGF-1 in human myocytes [15] and moreover, circulating Irisin levels have been positively correlated with IGF-1 in humans [13]. Taken together, these data suggest that the improved musculature and reduced fibrosis noted in *mdx* mice in the present study may be due to elevated IGF-1 levels. However, further work will need to be performed to validate this hypothesis.

Since Irisin is an exercise induced myokine and is found in significantly higher concentrations post-exercise [12], and given the pro-myogenic function of Irisin revealed in the current study, we propose that Irisin could be the molecule, which mediates the beneficial effects of exercise on skeletal muscle, without the associated muscle damage due to excessive physical exertion. This is particularly relevant in conditions such as DMD where exercise has been shown to exacerbate disease symptoms [38]. Overall, the results presented in this report show that Irisin may be a promising therapeutic target for the intervention of muscle dystrophy.

## MATERIALS AND METHODS

### Purification of recombinant irisin protein

cDNA corresponding to murine Irisin (amino acids 29-140 of FNDC5) was PCR amplified and cloned in frame into the pET-16b T7 expression vector, as per standard molecular biological techniques, and the resulting plasmid was transformed into the BL21 strain of *E.coli* (Agilent Technologies, USA). The recombinant murine Irisin protein was induced and purified to homogeneity using Ni-Agarose resin under native conditions. Purified Irisin protein was dialyzed for three hours against buffer containing 50mM Tris-HCl (pH 8.0), 500 mM NaCl and 10% Glycerol (dialysis buffer, DB) with three changes. Endotoxin in the purified Irisin protein was estimated using the ToxinSensor Chromogenic LAL kit, according to the protocol supplied with the kit.

### Western blot analysis

In order to extract proteins, muscle tissue (thirty milligrams) homogenates were prepared in RIPA buffer (1x Phosphate buffered saline, 1% IGEPAL CA-630 (v/v), 0.1% Sodium dodecyl sulfate (w/v), 0.5% Sodium deoxycholate (w/v)), 50mM Sodium fluoride) using the Tissue Lyser II disruptor (Qiagen, USA) at 30Hz (3 × 1 minute). After centrifugation of homogenized muscle tissue for 10 minutes at 12,000 rpm, supernatant was used for protein estimation and western blot analysis. 50μg of total protein was separated by SDS-PAGE (8% Tris-Glycine acrylamide gel) and transferred to nitrocellulose membrane by electroblotting. Membranes were stained with Ponceau S (Fluka, Sigma-Aldrich, USA) to confirm equal loading of protein samples. The membranes were blocked for 1 hour at room temperature with 5% milk (Sigma, USA) in TBST and subsequently incubated with either anti-Utrophin (1:200, Santa Cruz, SC-33700) or anti-Vinculin (1:5000, Sigma Aldrich, V9131) primary antibodies overnight at 4°C. The membranes were washed with TBST (3 × 10 minutes), followed by secondary antibody incubation for 1 hour at room temperature (RT). Secondary antibodies used in this paper were anti-mouse HRP (1:5000, Bio-Rad USA). Following secondary antibody incubations, membranes were washed as stated above and HRP activity was detected using Western Lighting Chemiluminescence Reagent Plus (NEL104; PerkinElmer Life Sciences, USA). Bands were detected using the ChemiDoc Touch Imaging System (BioRad, USA) and were analyzed using Image Lab software (BioRad, USA).

### Animal care and treatment

Male *mdx* mice were bred in the Nanyang Technological University (NTU) animal house, Singapore. Animal experiments were performed according to the protocols approved by the NTU Institutional Animal Care & Use Committee (IACUC), Singapore. Mice were housed at a constant temperature of 20°C under an artificial 12h light and 12h dark cycle with *ad libitum* access to standard chow diet and water at NTU animal house. To monitor the effect of Irisin on dystrophic muscle growth, three trials were performed. In the first trial, 3-week-old *mdx* mice were injected with either recombinant Irisin protein (2.5μg of Irisin per gram of body weight) or DB for two weeks, three times-a-week and all hind limb muscles were collected for extensive characterization of the *mdx* phenotype and the role of Irisin in *mdx* mice. Cross sectional area (CSA) and extent of muscle fiber damage was analyzed. A second trial was performed in order to assess the CSA and fibrotic tissue accumulation by injecting 6-week-old *mdx* mice with either recombinant Irisin protein (2.5μg of Irisin per gram of body weight) or DB three times-a-week for a total of 2 weeks. In the final trial, 4-week-old *mdx* mice were injected intraperitoneally (IP) with either recombinant Irisin protein (2.5μg of Irisin per gram of body weight) or DB three times-a-week for a total of 4 weeks to analyze if a longer period of treatment enhances muscle weight and grip strength. Grip strength was measured just prior to the dissection in the final trial. Six readings were obtained for each mouse with a 30 seconds rest between each reading. The highest three values were used to calculate the average grip strength for each mouse in both the DB and Irisin injected groups. Measurements were recorded in Newtons (N). Food intake was measured before every injection. At the end of each trial, mice were euthanized by CO_2_ asphyxiation and various hind limb muscles were harvested, weighed and stored for further experiments. The weights of the hind limb muscles were normalized to the tibia lengths of respective mice. TA muscle tissue was embedded in O.C.T (Sakura Finetek, USA), and stored at -80°C.

### Muscle histology

For performing muscle histology, 10μm TA muscle transverse sections were generated from the midbelly region of O.C.T embedded muscle tissue using a Cryostat (Leica CM 1950, Germany). The muscle sections were stained with Gill's Haematoxylin followed by 1% Eosin (Merck) (H&E). The cross sectional area of 200 muscle fibers from 5 random fields (1000 fibers) were analyzed per section. Myofibers that contained centrally placed nuclei were counted and normalized as a percentage of the total number of myofibers in the muscle section using ImageJ software (National Institutes of Health, USA). Necrotic muscle fibers were assessed based on the areas of fragmented sarcoplasm, influx of mononucleated cells and hyper-contracted muscle fibers. Muscle sections were tiled using the Leica CTR 6500 microscope, equipped with the Leica DFC 420 camera and Image Pro Plus software (Media Cybernetics, Bethesda, MD) and a 10x objective. Representative images were taken at a higher magnification (20x objective).

### Measurement of fibrosis

Muscle sections were stained using the Sirius Red/Fast Green collagen staining kit, as per the manufacturer's protocol (Chondrex Inc, WA). Muscle sections were immersed in dye solution for 30 minutes at room temperature and excess dye was washed off with distilled water. Stained sections were then dried and mounted using DPX mounting medium (Merck, USA). The tissue sections were photographed and tiled using the Leica CTR 6500 microscope, equipped with the Leica DFC 420 camera and Image Pro Plus software (Media Cybernetics, Bethesda, MD) and a 10x objective. Representative images were taken at a higher magnification (40x objective). The extent of collagen deposition (red stain) was quantified by determining the total area that was stained red across the entire muscle cross section. The total area stained red was normalized to the total TA muscle section area and expressed as a percentage, to represent percentage fibrosis.

### Immunohistochemical staining of muscle sections

To detect IgM accumulation in muscle fibers, muscle sections were fixed with ice-cold acetone for 2 minutes and washed 3 times with 1xPBS for 5 minutes each. Blocking was performed for 1 hour at room temperature with 1% BSA and 5% horse serum in 1x PBS. Sections were subsequently incubated with Goat anti-mouse IgM-FITC conjugated antibody (1:100, Sigma Aldrich, F9259) for 1 hour and washed again 3 times with 1xPBS for 5 minutes each. Sections were mounted with Slowfade Gold anti fade reagent (Molecular Probes) and visualized and tiled using the Leica CTR 6500 microscope, equipped with the Leica DFC 420 camera and Image Pro Plus software (Media Cybernetics, Bethesda, MD) and a 10x objective. Representative images were taken at a higher magnification (20x objective). Stained samples were analyzed and IgM positive fibers were counted, with the number of IgM positive myofibers expressed as a percentage of total muscle fiber area.

### Injection of evans blue dye

5-week-old male *mdx* mice were injected intraperitoneally with a solution of 1% Evans Blue Dye (EBD) at a volume of 10μl per gram body weight 24h prior to hind limb muscle collection. The extent of EBD accumulation was assessed based on the area of the muscle section that displayed red fluorescence, expressed as a percentage of the total area of the muscle section. Sections were visualized and tiled using the Leica CTR 6500 microscope equipped with the Leica DFC 420 camera and Image Pro Plus software (Media Cybernetics, Bethesda, MD) and a 10x objective. Representative images were taken at a higher magnification (20x objective).

### Creatine kinase assay

Creatine kinase (CK) assay was performed to assess the extent of muscle damage in the *mdx* mice injected with either DB or Irisin. CK is usually present in skeletal muscle, but leaks into the serum upon muscle fiber damage. CK assay was performed using a Creatine Kinase Activity Assay Kit (KA3766), according to the manufacturer's protocol. CK activity was measured in a clear bottom 96-well plate at 450 nm using a Microplate Spectrophotometer (BioRad, USA) and the MPM 6 software. Calculations were performed according to the manufacturer's instructions and creatine kinase values were represented as mU/mL.

### Statistical analysis

Two-tailed Student's t-test was used to compare differences between two groups. Equal variance between the two groups was determined by performing an F test on data prior to performing the Student's t-test. If the F-value was smaller than F-critical (Type 2 error), the variance between the two groups was deemed equal. However, when the F-value was greater than F-critical values (Type 3 error) then the variance was deemed to be unequal. Results were considered significant at p< 0.05 (^*^), p< 0.01 (^**^) and p< 0.001(^***^). Data is presented as mean ± SEM.
